# Supplementary material for: Altered NMDAR signaling underlies autistic-like features in mouse models of CDKL5 deficiency disorder
Source: Nat Commun. 2019 Jun 14;10:2655. doi: 10.1038/s41467-019-10689-w (PMC6572855; doi:10.1038/s41467-019-10689-w)
Supplement: Supplementary file 1 — Supplementary Information [file 41467_2019_10689_MOESM1_ESM.docx]

Supplemental Figures

Tang et al.


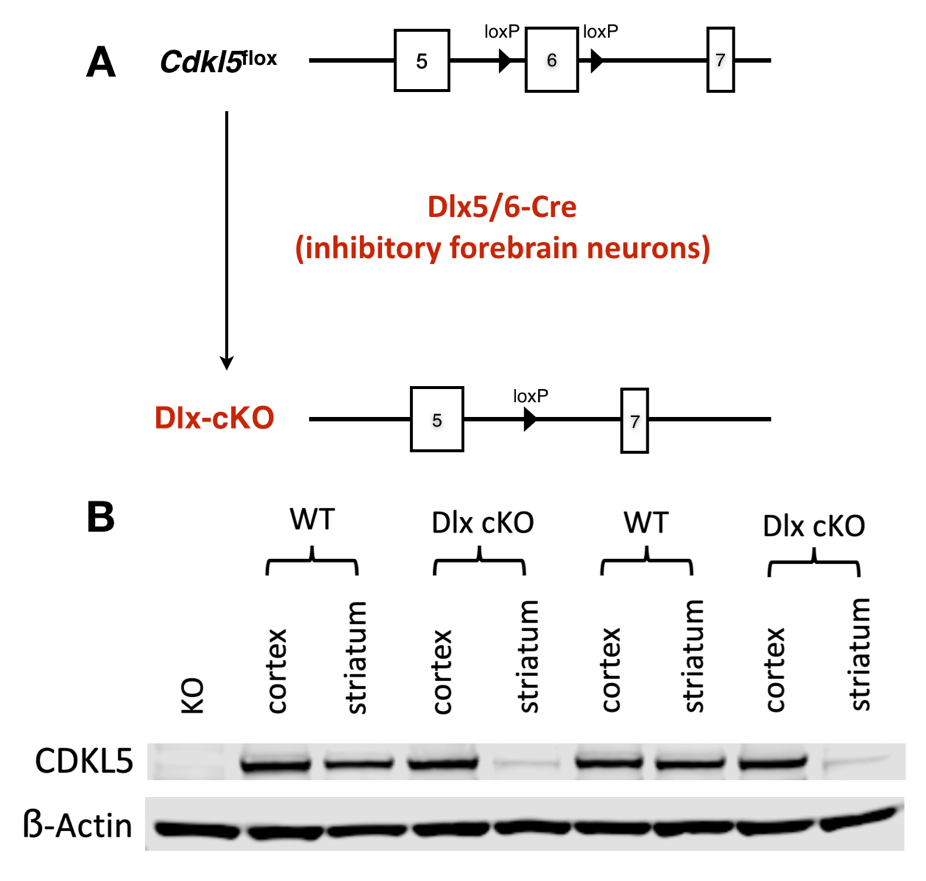


**Supplemental Figure 1. Generation and validation of mice lacking CDKL5 selectively in forebrain GABAergic neurons (Dlx-cKO).**

(A) Schematic representing the generation of Dlx-cKO mice using a conditional *Cdkl5* exon 6-floxed Cdkl5 mouse line (Tang et al., 2017) and the Dlx5/6-Cre driver (Monory et al., 2006). (B) Validation of the cell type-specificity of CDKL5 protein knockout using microdissected brain tissue. Two pairs of WT and Dlx-cKO littermates were used. In the cortex, a region enriched for glutamatergic neurons, Dlx-cKO mice show comparable amounts of CDKL5 protein to WT. In the striatum, a region enriched for GABAergic neurons, Dlx-cKO mice show reduced CDKL5 protein in comparison to WT. The residual CDKL5 protein likely originates from non-GABAergic cells in this brain region.


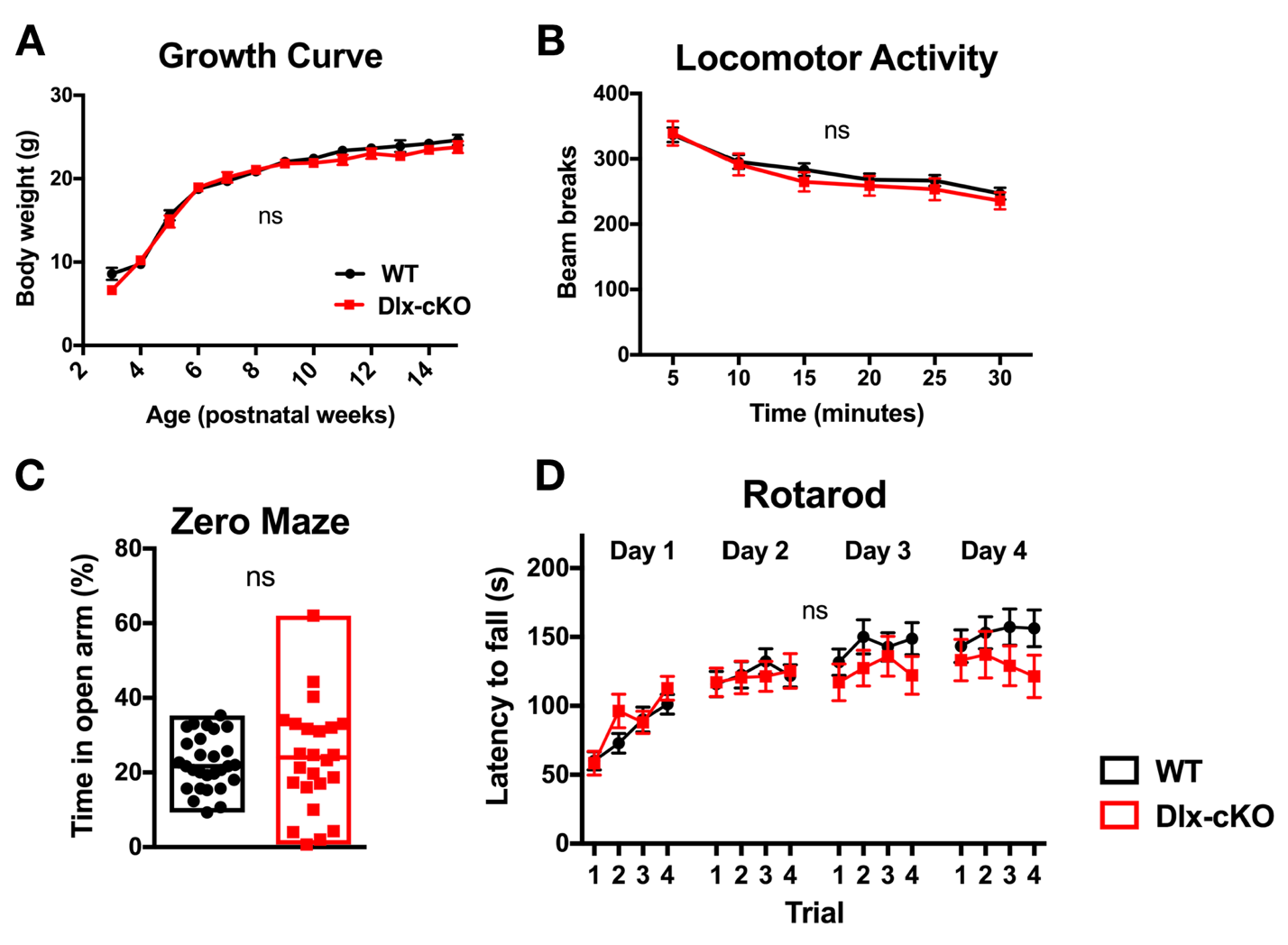


**Supplemental Figure 2. Dlx-cKO mice show unaltered growth, locomotion, anxiety-related behavior, and motor coordination.**

(A) Unaltered body weight of Dlx-cKO mice at various ages (WT, n = 4-36 mice / time point; Dlx-cKO, 14-29 mice / time point; unpaired t-tests with Holm-Sidak’s correction for multiple comparisons). (B) Dlx-cKO mice show unaltered home-cage locomotion on the beam break assay (WT, n = 43, Dlx-cKO, n = 32; one-way repeated-measures ANOVA). (C) Dlx-cKO mice, show unaltered anxiety-related behavior, as assessed by the percent time spent in open arms, on the elevated zero maze assay (WT, n = 28, Dlx-cKO, n = 24; unpaired t-test). (D) Dlx-cKO mice show unaltered motor coordination and learning on the Rotarod assay (WT, n = 39, Dlx-cKO, n = 23; one-way repeated-measures ANOVA). Error bars s.e.m. Source data are provided as a Source Data file.


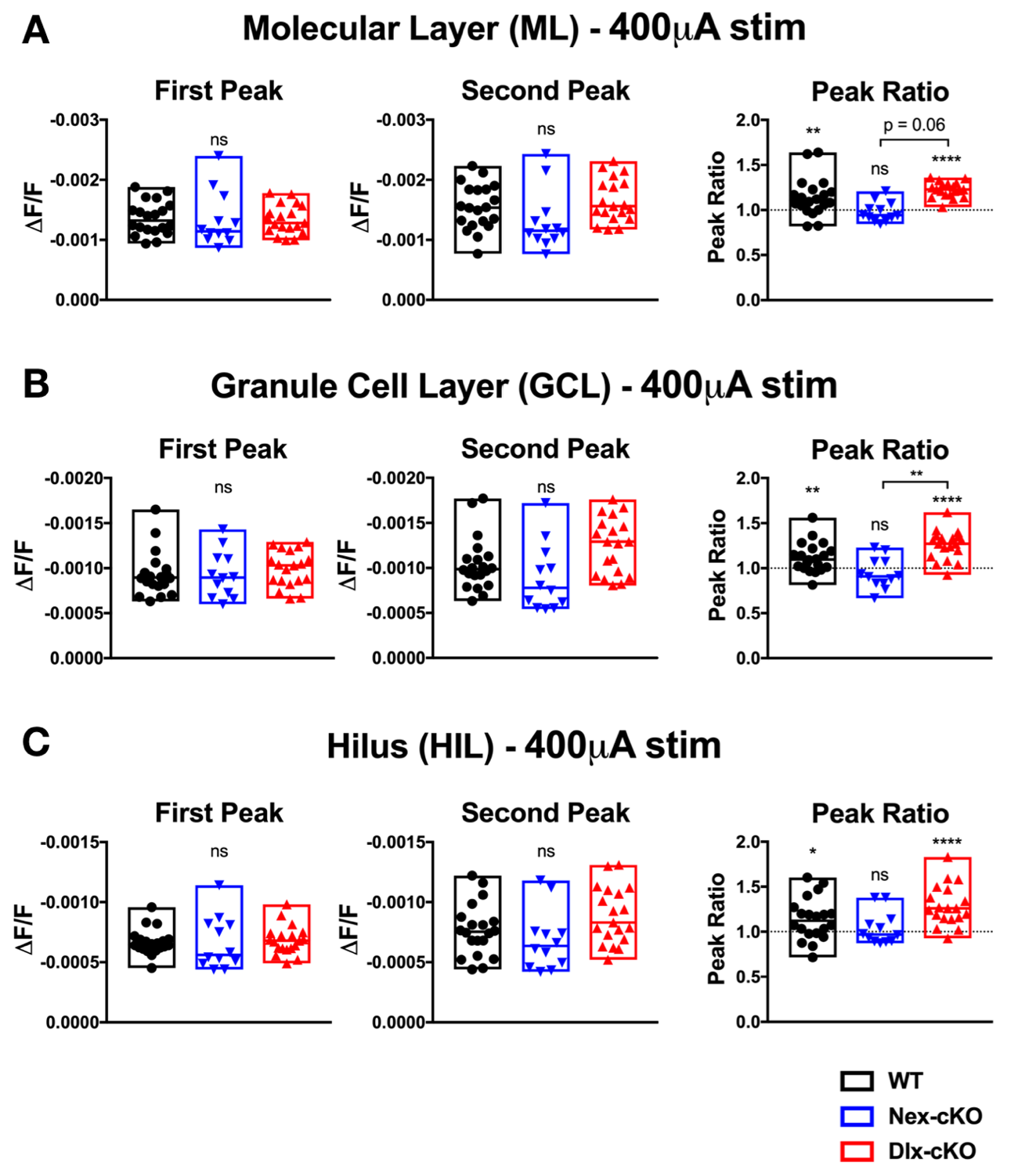


**Supplemental Figure 3. VSDI responses in regions of the dentate gyrus at 400μA stimulation intensity.**

(A-C) Peak responses are not significantly different between WT, Nex-cKO, and Dlx-cKO (linear mixed effect analysis). In all three regions (ML, GCL, and HIL) at this higher stimulation intensity, Dlx-cKO show significantly increased facilitation, whereas Nex-cKO shows no significant facilitation or depression (one-sample t-test or Wilcoxon signed-rank test, two-tailed). In the GCL, Dlx-cKO also showed significantly increased paired-pulse ratio in comparison to Nex-cKO (linear mixed effect analysis with Tukey’s correction for multiple comparisons). *p<0.05, **p<0.01, ***p<0.001, and ****p<0.0001. Source data are provided as a Source Data file.


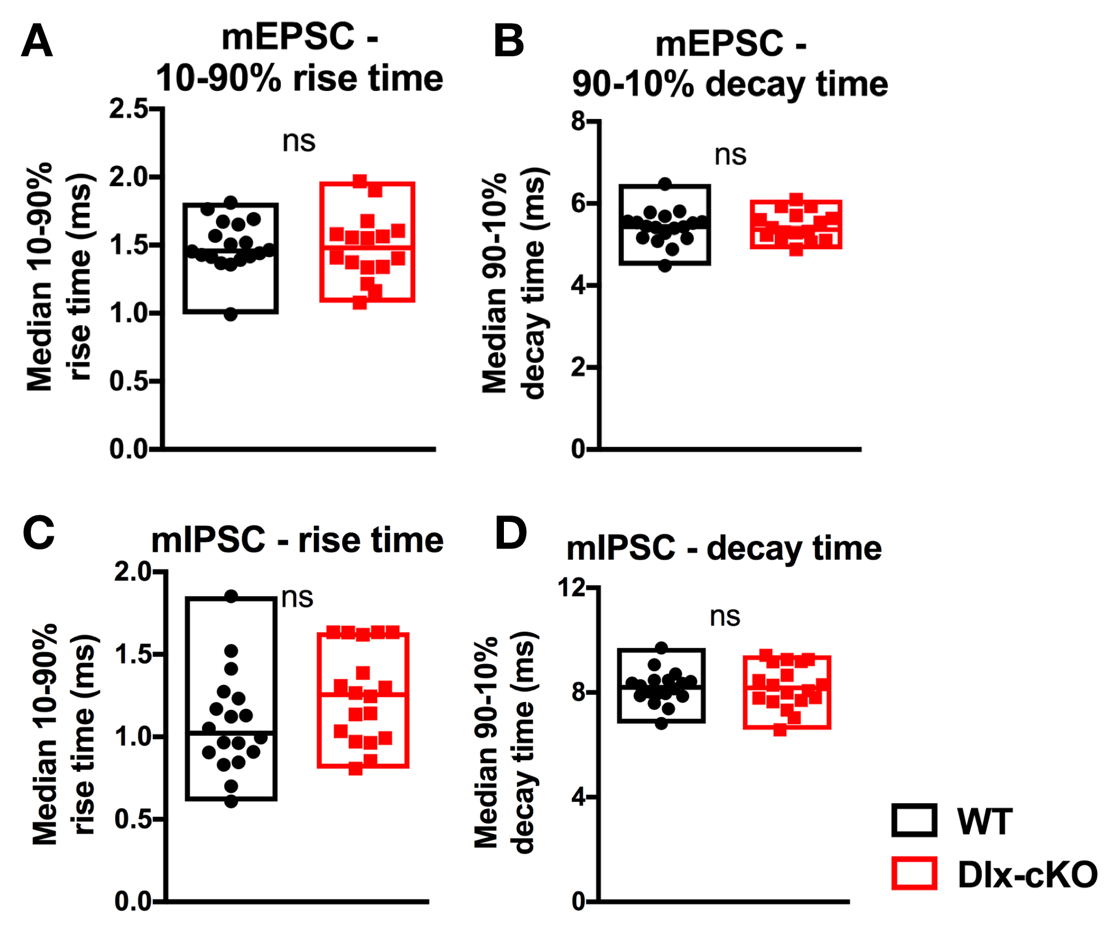


**Supplemental Figure 4. Unaltered mEPSC and mIPSC kinetics in Dlx-cKO mice.**

(A) The median 10-90% rise time of mEPSCs is unaltered in Dlx-cKO mice (linear mixed effect analysis). (B) The median 90-10% decay times of mEPSCs is unaltered in Dlx-cKO mice (linear mixed effect analysis). (C) The median 10-90% rise time of mIPSCs is unaltered in Dlx-cKO mice (linear mixed effect analysis). (D) The median 90-10% decay times of mIPSCs is unaltered in Dlx-cKO mice (linear mixed effect analysis). Source data are provided as a Source Data file.


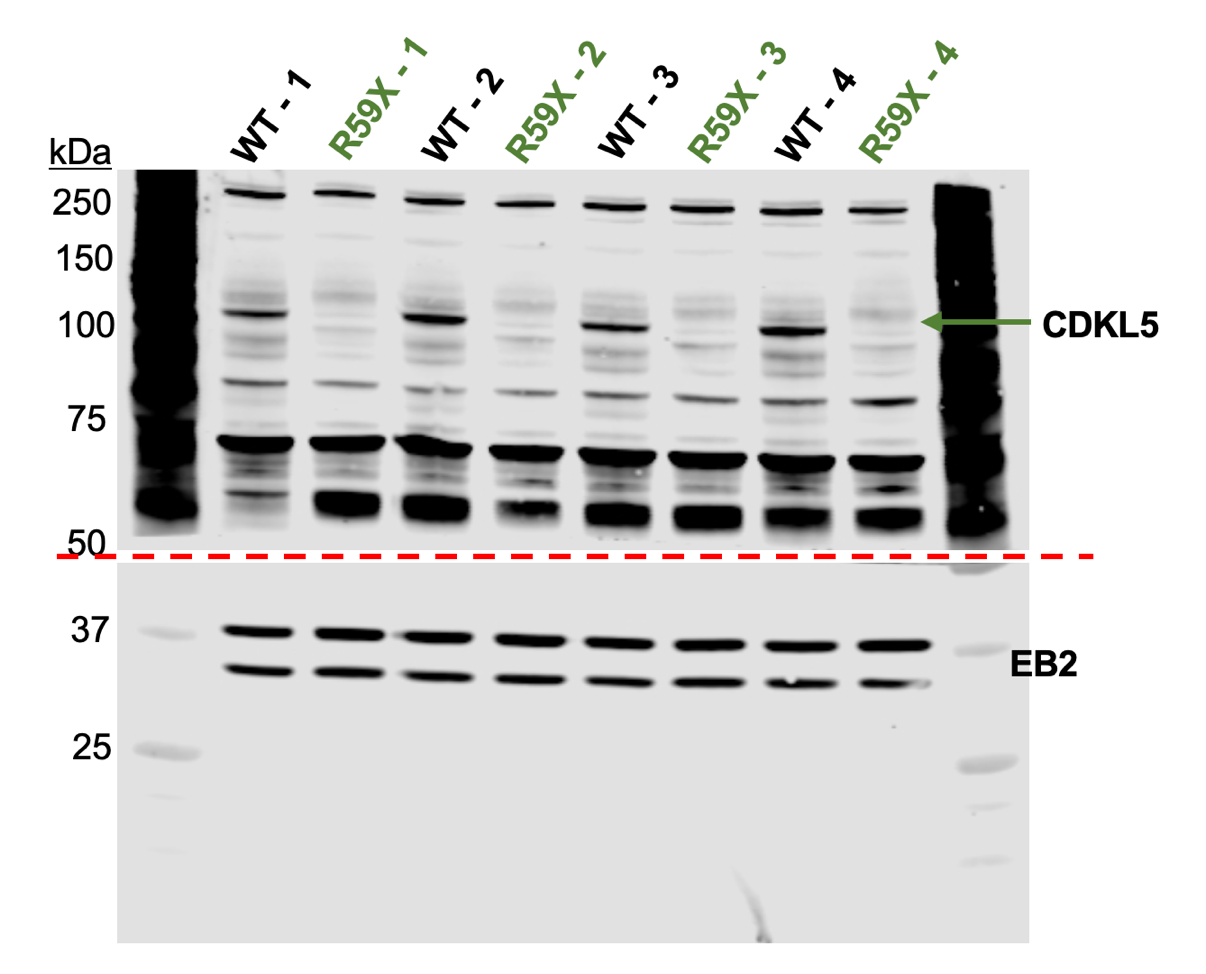


**Supplemental Figure 5. Loss of full-length CDKL5 protein in R59X knock-in mice.**

Loss of full-length CDKL5 protein in R59X mice, shown with western blots of dissected whole cortex lysate in four pairs of WT and R59X littermates. Green arrow indicates band corresponding to CDKL5 (note a non-specific band running at approximately the same size). Total EB2 protein was used as a loading control.

**Supplemental Figure 6. Full scan western blots.** The first blot is for supplemental figure 1B. The others are for postsynaptic density (PSD) protein preparations (Figures 4A and 6B). On each blot, the loading order consists of alternating lanes of WT and Dlx-cKO/R59X. Biorad Precision Plus Protein Standard (All-Blue) was used to demonstrate 10-250kD range. Some blots were cut and probed with multiple antibodies (as indicated). If blot was cut, red dotted line indicates position of cut. Note that some blots are shown at varying exposures to better visualize specific proteins of interest. Black dotted lines indicated cropped regions used to demonstrate representative bands in main article figures.

**Suppl Figure 1B (full scan blot)**


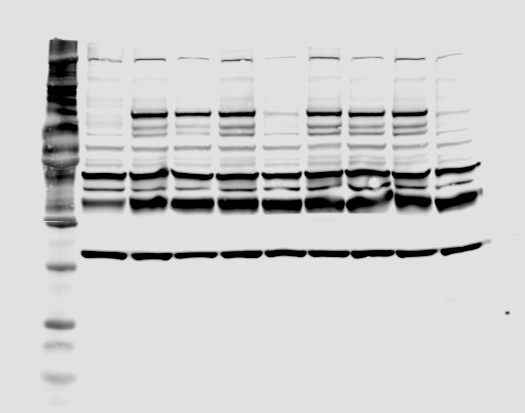


CDKL5 (105kDa)

ß-Actin (42kDa)

KO

cortex

striatum

cortex

striatum

cortex

striatum

cortex

striatum

**WT**

250

150

100

75

50

37

25

kDa

**Dlx-cKO**

**WT**

**Dlx-cKO**


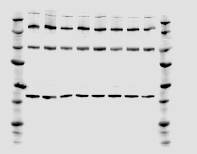


250

150

100

75

50

37

25

kDa

GluN2A (175kDa)

GluA1 (100kDa)

Actin (42kDa)

**Figure 4A (full scan blots)**

**Dlx-cKO**

**Dlx-cKO**

**WT**

**Dlx-cKO**

**WT**

**WT**

**WT**

**Dlx-cKO**


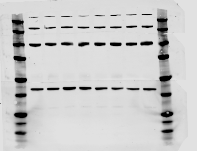


GluN2A (175kDa)

GluA1 (100kDa)

Actin (42kDa)

250

150

100

75

50

37

25

kDa

**Dlx-cKO**

**Dlx-cKO**

**WT**

**Dlx-cKO**

**WT**

**WT**

**WT**

**Dlx-cKO**


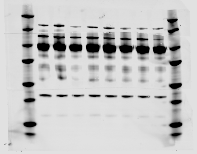

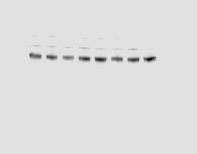

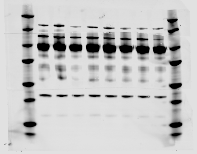


GluN2B (175kDa)

GluA2 (100kDa)

Actin (42kDa)

250

150

100

75

50

37

25

kDa

**Dlx-cKO**

**Dlx-cKO**

**WT**

**Dlx-cKO**

**WT**

**WT**

**WT**

**Dlx-cKO**


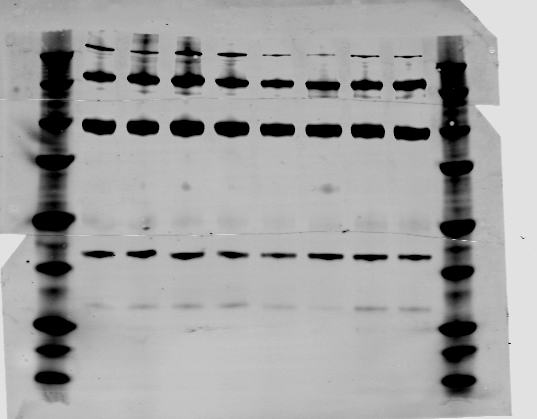


250

150

100

75

50

37

25

kDa

GluN2A (175kDa)

GluA1 (100kDa)

Actin (42kDa)

**Dlx-cKO**

**Dlx-cKO**

**WT**

**Dlx-cKO**

**WT**

**WT**

**WT**

**Dlx-cKO**


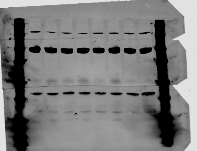

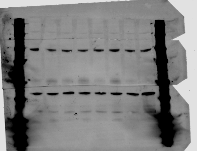


250

150

100

75

50

37

25

kDa

GluN2B (175kDa)

GluA2 (100kDa)

Actin (42kDa)

**Dlx-cKO**

**Dlx-cKO**

**WT**

**Dlx-cKO**

**WT**

**WT**

**WT**

**Dlx-cKO**

250

150

100

75

50

37

25

kDa


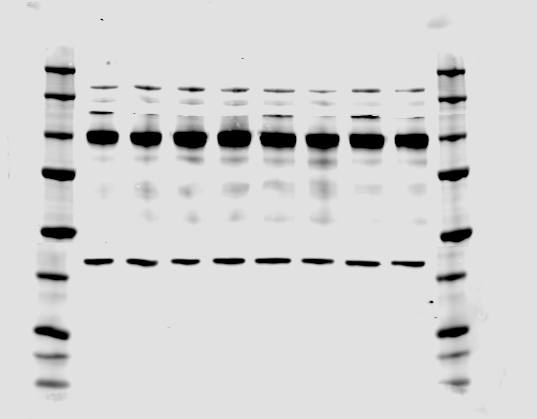

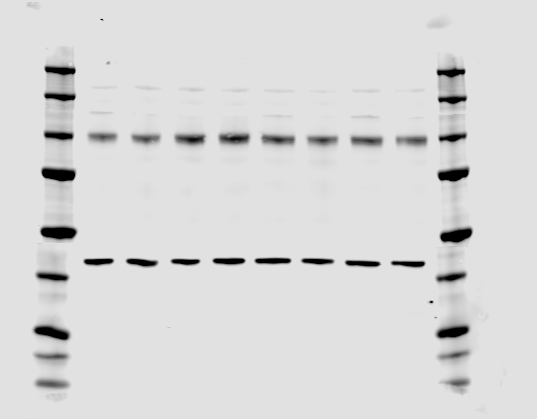


GluN2B (175kDa)

GluA2 (100kDa)

Actin (42kDa)

**Dlx-cKO**

**Dlx-cKO**

**WT**

**Dlx-cKO**

**WT**

**WT**

**WT**

**Dlx-cKO**


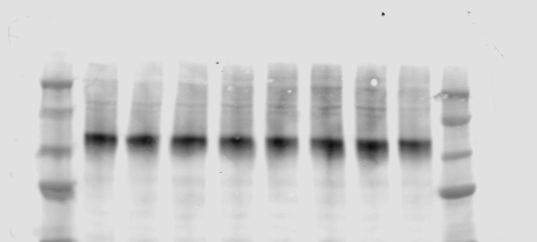

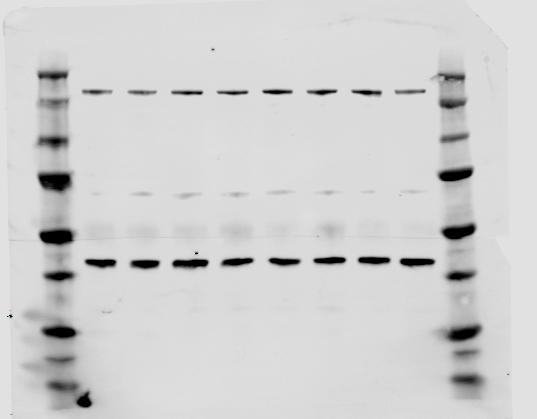


250

150

100

75

50

37

25

kDa

GluN1 (105kDa)

Actin (42kDa)

**Dlx-cKO**

**Dlx-cKO**

**WT**

**Dlx-cKO**

**WT**

**WT**

**WT**

**Dlx-cKO**

250

150

100

37

25

kDa

GluN1 (105kDa)

Actin (42kDa)


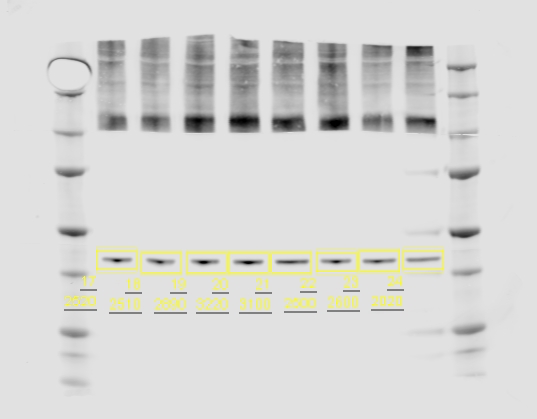

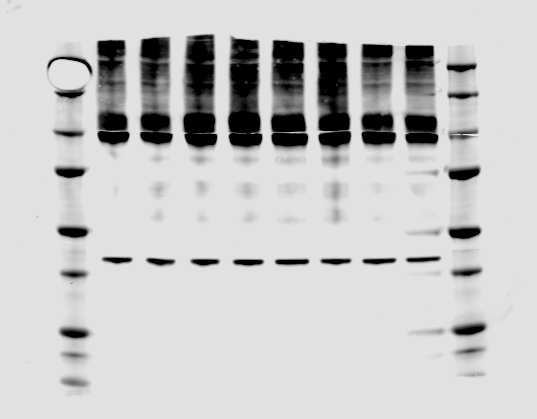


**Dlx-cKO**

**Dlx-cKO**

**WT**

**Dlx-cKO**

**WT**

**WT**

**WT**

**Dlx-cKO**


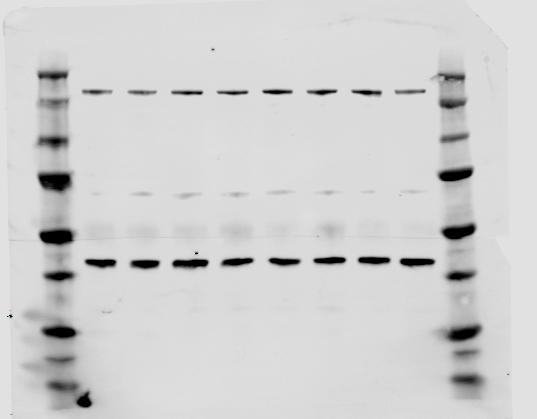


250

150

100

75

50

37

25

kDa

GluN2B (175kDa)

Actin (42kDa)

same blot reprobed

**Dlx-cKO**

**Dlx-cKO**

**WT**

**Dlx-cKO**

**WT**

**WT**

**WT**

**Dlx-cKO**


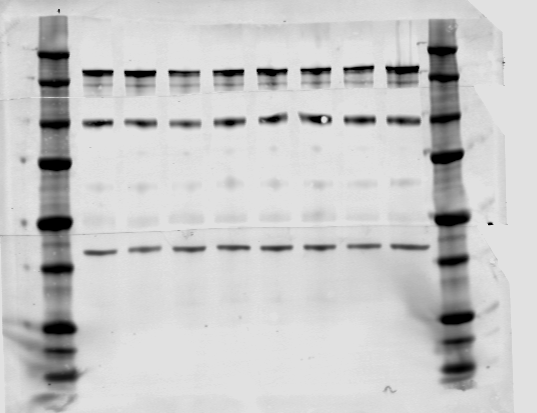


250

150

100

75

50

37

25

kDa

GluA1 (100kDa)

GluN2A (175kDa)

Actin (42kDa)

**R59X**

**R59X**

**R59X**

**R59X**

**WT**

**WT**

**WT**

**WT**

**Figure 6B (full scan blots)**


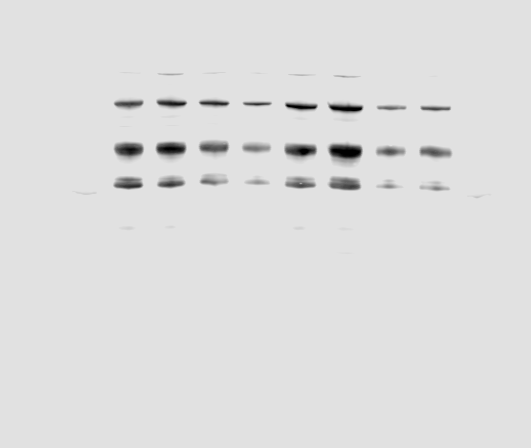

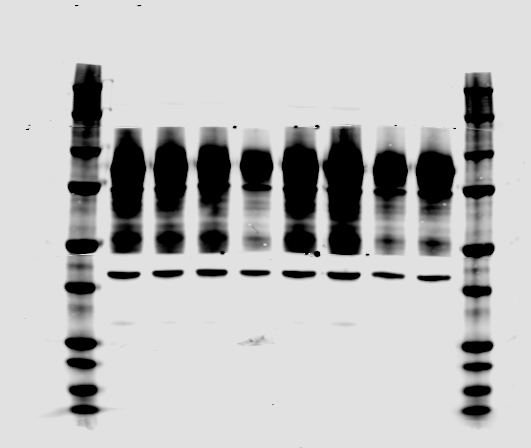


250

150

100

75

50

37

25

kDa

GluN2A (175kDa)

GluA1 (100kDa)

Actin (42kDa)

**R59X**

**R59X**

**R59X**

**R59X**

**WT**

**WT**

**WT**

**WT**


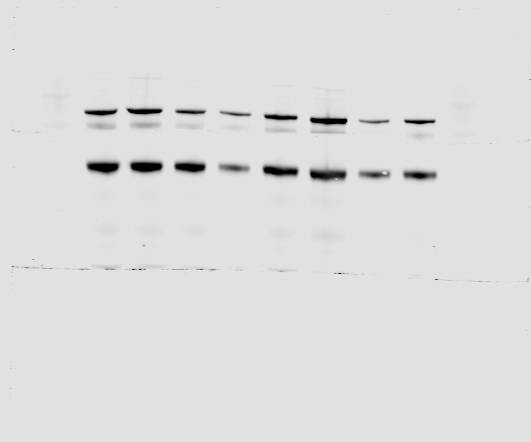

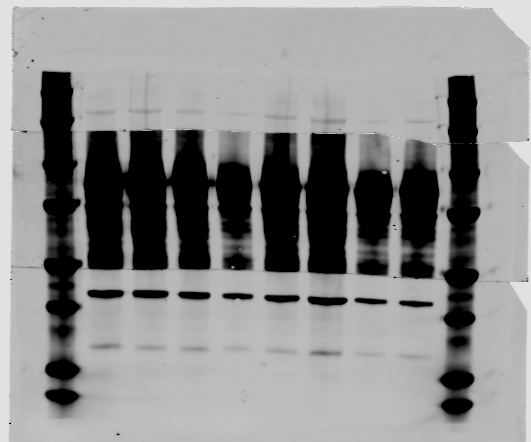


250

150

100

75

50

37

25

kDa

GluN2B (175kDa)

GluA1 (100kDa)

Actin (42kDa)

**R59X**

**R59X**

**R59X**

**R59X**

**WT**

**WT**

**WT**

**WT**


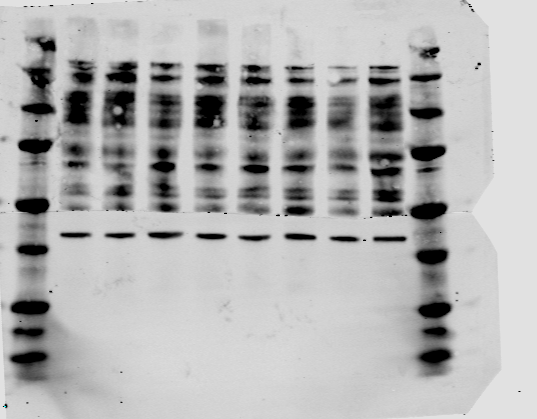


250

150

100

75

50

37

25

kDa

GluN2B (175kDa)

Actin (42kDa)

**R59X**

**R59X**

**R59X**

**R59X**

**WT**

**WT**

**WT**

**WT**

250

150

100

75

50

37

25

kDa


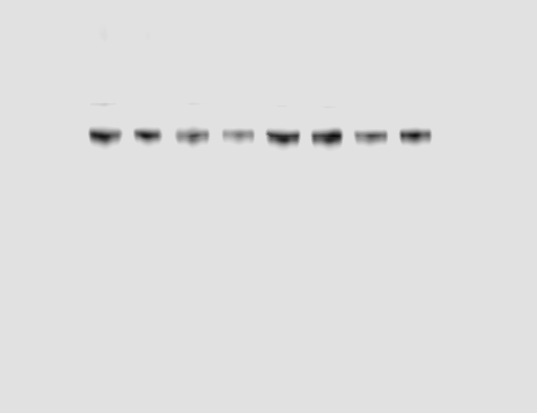

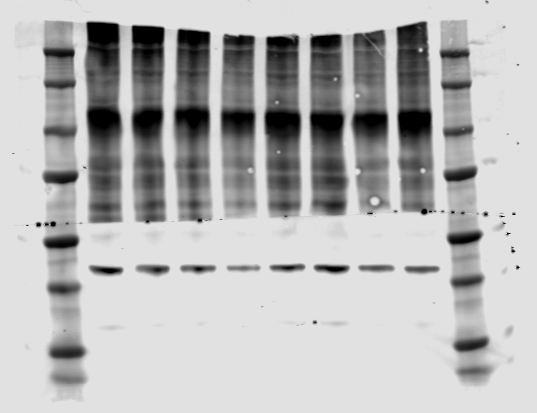


GluA2 (100kDa)

Actin (42kDa)

**R59X**

**R59X**

**R59X**

**R59X**

**WT**

**WT**

**WT**

**WT**


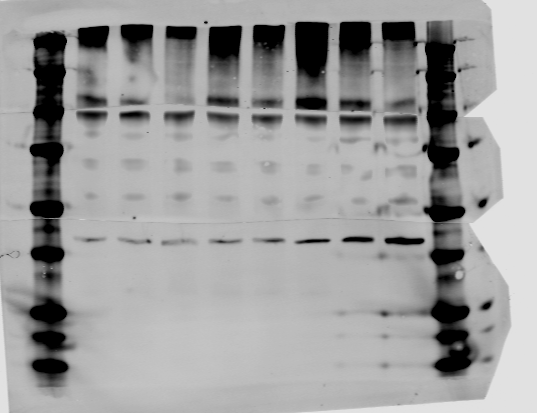


100

75

50

37

25

kDa

GluA2 (100kDa)

Actin (42kDa)

**R59X**

**R59X**

**R59X**

**R59X**

**WT**

**WT**

**WT**

**WT**


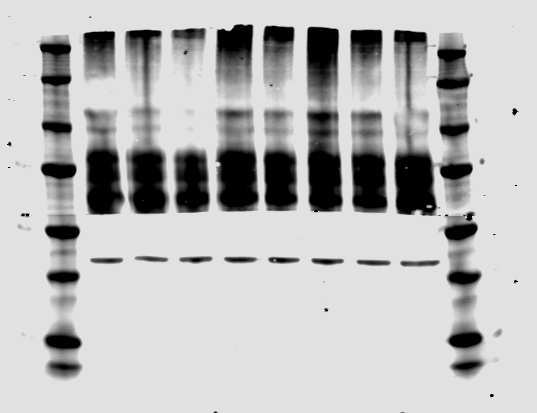


GluN1 (105kDa)

Actin (42kDa)

250

150

100

75

50

37

25

kDa

**R59X**

**R59X**

**R59X**

**R59X**

**WT**

**WT**

**WT**

**WT**


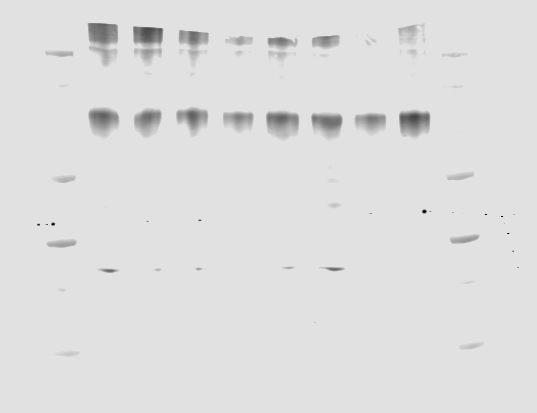

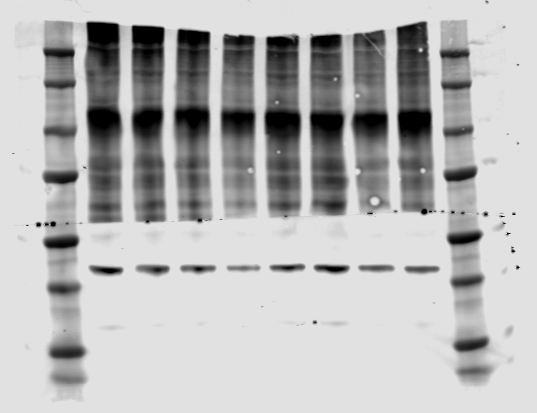


GluN1 (105kDa)

Actin (42kDa)

250

150

100

75

50

37

25

kDa

**R59X**

**R59X**

**R59X**

**R59X**

**WT**

**WT**

**WT**

**WT**
