# Supplementary material for: Altered NMDAR signaling underlies autistic-like features in mouse models of CDKL5 deficiency disorder
Source: Nat Commun. 2019 Jun 14;10:2655. doi: 10.1038/s41467-019-10689-w (PMC6572855; doi:10.1038/s41467-019-10689-w)
Supplement: Supplementary file 3 — Reporting Summary [file 41467_2019_10689_MOESM3_ESM.pdf]

nature research

Corresponding author(s): Douglas Coulter, Zhaolan Zhou

Last updated by author(s): May 15, 2019

Reporting Summary

Nature Research wishes to improve the reproducibility of the work that we publish. This form provides structure for consistency and transparency in reporting. For further information on Nature Research policies, see Authors & Referees and the Editorial Policy Checklist.

Statistics

For all statistical analyses, confirm that the following items are present in the figure legend, table legend, main text, or Methods section.

n/a	Confirmed	
☐	☒	The exact sample size (*n*) for each experimental group/condition, given as a discrete number and unit of measurement
☐	☒	A statement on whether measurements were taken from distinct samples or whether the same sample was measured repeatedly
☐	☒	The statistical test(s) used AND whether they are one- or two-sided<br>*Only common tests should be described solely by name; describe more complex techniques in the Methods section.*
☒	☐	A description of all covariates tested
☐	☒	A description of any assumptions or corrections, such as tests of normality and adjustment for multiple comparisons
☐	☒	A full description of the statistical parameters including central tendency (e.g. means) or other basic estimates (e.g. regression coefficient) AND variation (e.g. standard deviation) or associated estimates of uncertainty (e.g. confidence intervals)
☐	☒	For null hypothesis testing, the test statistic (e.g. *F*, *t*, *r*) with confidence intervals, effect sizes, degrees of freedom and *P* value noted<br>*Give P values as exact values whenever suitable.*
☒	☐	For Bayesian analysis, information on the choice of priors and Markov chain Monte Carlo settings
☒	☐	For hierarchical and complex designs, identification of the appropriate level for tests and full reporting of outcomes
☒	☐	Estimates of effect sizes (e.g. Cohen's *d*, Pearson's *r*), indicating how they were calculated

Our web collection on statistics for biologists contains articles on many of the points above.

Software and code

Policy information about availability of computer code

Data collection	Behavioral, electrophysiology, or calcium imaging data was acquired using pCLAMP10 (Axon Instruments, Molecular Devices), 80x80 CCD camera (NeuroCCD; RedShirt Imaging), and SMART Video Tracking software (3.0)
Data analysis	Behavior, electrophysiology, or calcium imaging data was analyzed using IGOR (Wavemetrics), R (The R Project for Statistical Computing; 3.4.4), and Prism (GraphPad, Version7)

For manuscripts utilizing custom algorithms or software that are central to the research but not yet described in published literature, software must be made available to editors/reviewers. We strongly encourage code deposition in a community repository (e.g. GitHub). See the Nature Research guidelines for submitting code & software for further information.

Data

Policy information about availability of data

All manuscripts must include a data availability statement. This statement should provide the following information, where applicable:

- Accession codes, unique identifiers, or web links for publicly available datasets
- A list of figures that have associated raw data
- A description of any restrictions on data availability

Raw data for Figures 1-7 and Supplemental Figures 2-4 is provided as a Source Data file. Original full-scan western blots are included in Supplemental Figure 6.

Field-specific reporting

Please select the one below that is the best fit for your research. If you are not sure, read the appropriate sections before making your selection.

☒ Life sciences ☐ Behavioural & social sciences ☐ Ecological, evolutionary & environmental sciences

Life sciences study design

All studies must disclose on these points even when the disclosure is negative.

Sample size	Sample sizes for behavioral experiments were based on previous published studies of Cdkl5 constitutive and conditional knockout mice. For behavioral assays involving drug administration, we used pilot behavioral cohorts to estimate effect sizes and then used similar sample sizes for saline- and drug-administered cohorts. The number of mice used were pre-determined prior to the start of each experiment with an n=6-20 for behavioral experiments, n=3-6 for electrophysiology and calcium imaging, and n=8-10 for protein biochemistry
Data exclusions	No data was excluded
Replication	Biological replicates were performed for all experiments (see sample sizes above)
Randomization	All mice were allocated into sex-matched, littermate-matched experimental groups
Blinding	All experiments were performed with the researcher blinded to genotype.

Reporting for specific materials, systems and methods

We require information from authors about some types of materials, experimental systems and methods used in many studies. Here, indicate whether each material, system or method listed is relevant to your study. If you are not sure if a list item applies to your research, read the appropriate section before selecting a response.

Materials & experimental systems

n/a	Involved in the study
☐	☒ Antibodies
☒	☐ Eukaryotic cell lines
☒	☐ Palaeontology
☐	☒ Animals and other organisms
☒	☐ Human research participants
☒	☐ Clinical data

Methods

n/a	Involved in the study
☒	☐ ChIP-seq
☒	☐ Flow cytometry
☒	☐ MRI-based neuroimaging

Antibodies

Antibodies used	anti-GluN1 (ThermoFisher, OMA1-04010); anti-GluN2A (Frontier Institute, AB_2571605); anti-GluN2B (Frontier Institute, AB_2571761); anti-GluA1 (Abcam, ab31232); anti-GluA2 (Abcam, ab133477); anti-beta-Actin (Abcam, ab8226); anti-GAPDH (Invitrogen, MA5-15738); anti-EB2 (Abcam, ab45767); anti-CDKL5 (Millipore Sigma, ABS402)
Validation	All antibodies are commercially available and have been tested in mice.

Animals and other organisms

Policy information about studies involving animals; ARRIVE guidelines recommended for reporting animal research

Laboratory animals	6-12 week old male mice (mus musculus) maintained in the C59BL/6J background
Wild animals	not applicable
Field-collected samples	not applicable
Ethics oversight	All animal experiments were performed according to protocols approved by the Institutional Animal Care and Use Committee at the University of Pennsylvania in accordance with the National Institutes of Health guidelines.

Note that full information on the approval of the study protocol must also be provided in the manuscript.
